# Shear bond strength of new and recycled orthodontic brackets to enamel surface: an *in-vitro* study

**DOI:** 10.3389/fdmed.2026.1978887

**Published:** 2026-10-02

**Authors:** Nadine Ali Abdallah, Ahmed A. Holiel, Aly Osman

**Affiliations:** 1 Department of Developmental Science, Faculty of Dentistry, Beirut Arab University, Beirut, Lebanon; 2 Department of Restorative Sciences, Faculty of Dentistry, Beirut Arab University, Beirut, Lebanon; 3 Conservative Dentistry Department, Faculty of Dentistry, Alexandria University, Alexandria, Egypt; 4 Developmental Sciences Department, Division of Orthodontics, Faculty of Dentistry, Beirut Arab University, Beirut, Lebanon

**Keywords:** air abrasion, ARI, direct flaming, recycled brackets, shear bond strength

## Abstract

**Background:**

Recycling brackets is an efficient method to reduce the cost of treatment and decrease waste to the environment. However, various recycling procedures can alter the base of the bracket, thus affecting the bond strength and clinical outcomes.

**Aim of the study:**

This study aimed to evaluate the SBS of new and recycled stainless steel orthodontic brackets to enamel surfaces following different recycling procedures.

**Materials and methods:**

Fifty extracted human premolars were collected, bonded with stainless steel orthodontic brackets. The premolars were randomly divided into five groups, Group I (control, no recycling), Group II (air abrasion), Group III (silicon carbide stone), Group IV (direct flaming), and Group V (new brackets). Rebonding was performed using the same bonding procedure. All specimens were thermocycled between 5 °C and 55 °C for 3,000 cycles and tested for shear bond strength at a crosshead speed of 1 mm/min using a universal testing machine. The adhesive remnant index (ARI) was evaluated under a stereomicroscope.

**Results:**

Significant differences were found between groups (*p* < 0.001). The highest mean SBS was observed in the new brackets group followed by the air abrasion group Grinding and flaming groups showed the lowest values. Most specimens showed an ARI score of 3, indicating that a high amount of adhesive remained on the enamel surface after debonding.

**Conclusions:**

Air abrasion proved to be the most effective recycling technique, producing bond strength comparable to new brackets. Grinding and flaming resulted in lower SBS values and are less suitable for bracket recycling.

## Introduction

In orthodontics, there is a growing trend toward simplifying technical procedures to decrease treatment time and cost. One of the biggest challenges that may be encountered by the orthodontist, and that is troublesome for the dentist and patient, is bracket debonding (1).

Several factors can cause bond failure, like inadequate cleaning of the tooth surface, etching, isolation, bonding or curing, changes in PH in the oral cavity, masticatory forces, extreme temperatures, and low retentiveness of the bracket base. Negligence in any of these factors can increase the risk of bracket debonding because it affects the bond strength of the bracket. The location of failure may be between the adhesive and bracket, within the adhesive, or between the tooth and adhesive (2).

A bracket debonding could interfere with mechanics, extends appointments, and raises the total treatment costs and time to the patient and the clinic (3). Since bracket debonding may occur during routine chewing forces, improper isolation, or wire stresses, clinicians have considered recycling the debonded bracket as a feasible method, provided the bond's performance and enamel safety are not compromised (4).

The goal of bracket recycling is to get rid of any remaining adhesive from the base and make the surface retentive again. Airborne-particle abrasion (air abrasion), silicon-carbide stone grinding, and direct flaming are all common methods to accomplish this. Air abrasion micro-etches the base with aluminum-oxide particles to clean and re-texture the mesh. Grinding removes resin mechanically with a rotary stone. Flaming burns off adhesive quickly with direct heat (5). While these techniques are simple and accessible, they may modify the mesh pattern or surface chemistry of the base and thus influence the shear bond strength (SBS) and the failure pattern at debonding (6).

The optimal recycling technique in everyday practice needs to be predictable, quick and enamel safe. The SBS should be adequate to withstand the masticatory and orthodontic forces but permit controlled debonding without damaging the enamel (7). This applies to the Adhesive Remnant Index (ARI); the larger the index, the more likely it is to fail at the bracket adhesive interface, leaving more resin on the enamel, which may protect it, but combined with more clean-up (8).

The literature is heterogeneous despite numerous reports. Bracket brand and base design, air abrasion particle size and pressure, grinding speed and exposure time, flaming distance and flame time, bonding systems, and aging procedures also vary in research. Other studies do not use standardized thermocycling as well, which restricts their capacity to estimate fluctuations in intraoral temperature. Others lack a new-bracket comparison, which is needed to understand whether a recycling approach can indeed be comparable to fresh clinical performance (7).

To address these issues, the current *in-vitro* experiment provides a direct comparison of SBS of control and new brackets with recycled brackets subjected to three chairside procedures air abrasion, silicon-carbide stone grinding, as well as direct flaming.

By controlling key parameters and including both previously bonded and non-previously bonded teeth with new bracket groups, this study seeks to provide practical, parameter-specific guidance for clinicians who wish to recycle brackets chairside.

## Materials and methods

The study design consists of an experimental *in-vitro* study. Sample size was estimated *a priori* using Epitools (https://epitools.ausvet.com.au), applying the formula for comparison of two independent means as a basis for the primary pairwise comparison of interest. Input parameters were drawn from Wanderley et al. (2006) (8) who reported mean SBS values of 5.10 MPa (SD: 2.26 MPa) and 4.22 MPa (SD: 2.65 MPa) for new and recycled brackets, respectively (originally reported as 0.52 ± 0.23 and 0.43 ± 0.27 kgf/mm^2^, converted to MPa by multiplying by 9.807). These values correspond to a small standardized effect size (Cohen's d = 0.36), which, at *α* = 0.05 (two-sided) and 80% power, indicates a theoretical requirement of approximately 122 teeth per group. Recognizing that this represented an impractical sample size for an *in-vitro* study using extracted teeth, and that the effect observed in Wanderley et al. reflected a specific bonding protocol and methodology that differed from that of the present study, a practical sample of 10 teeth per group (50 total) was selected, consistent with comparable published *in-vitro* studies in the field (5, 9). *Post-hoc* power analysis based on the observed effect sizes confirmed that this sample was more than adequate: the overall Kruskal–Wallis test achieved an observed *η*^2^ of 0.570 (large effect), and individual pairwise comparisons yielded *post-hoc* power exceeding 99% for all significant pairs, indicating that the study was sufficiently powered to detect the differences observed.

The selected teeth fulfilled the following inclusion and exclusion criteria. Inclusion criteria: intact human premolars with fully formed roots; no cracks, caries, restorations, fluorosis, hypoplasia, or previous bonding. Exclusion criteria: teeth with visible structural defects, enamel fractures, craze lines, or any surface contamination that could not be removed by pumicing. Prior to bonding, all crowns were cleaned with non-fluoridated pumice and rinsed, then embedded with the buccal surface-oriented perpendicular to the load direction.

Following initial bonding, the specimens were divided into five experimental groups, each group consisting of ten premolars, with the groups divided according to the recycling method used (Figure 1).

**Figure 1:**
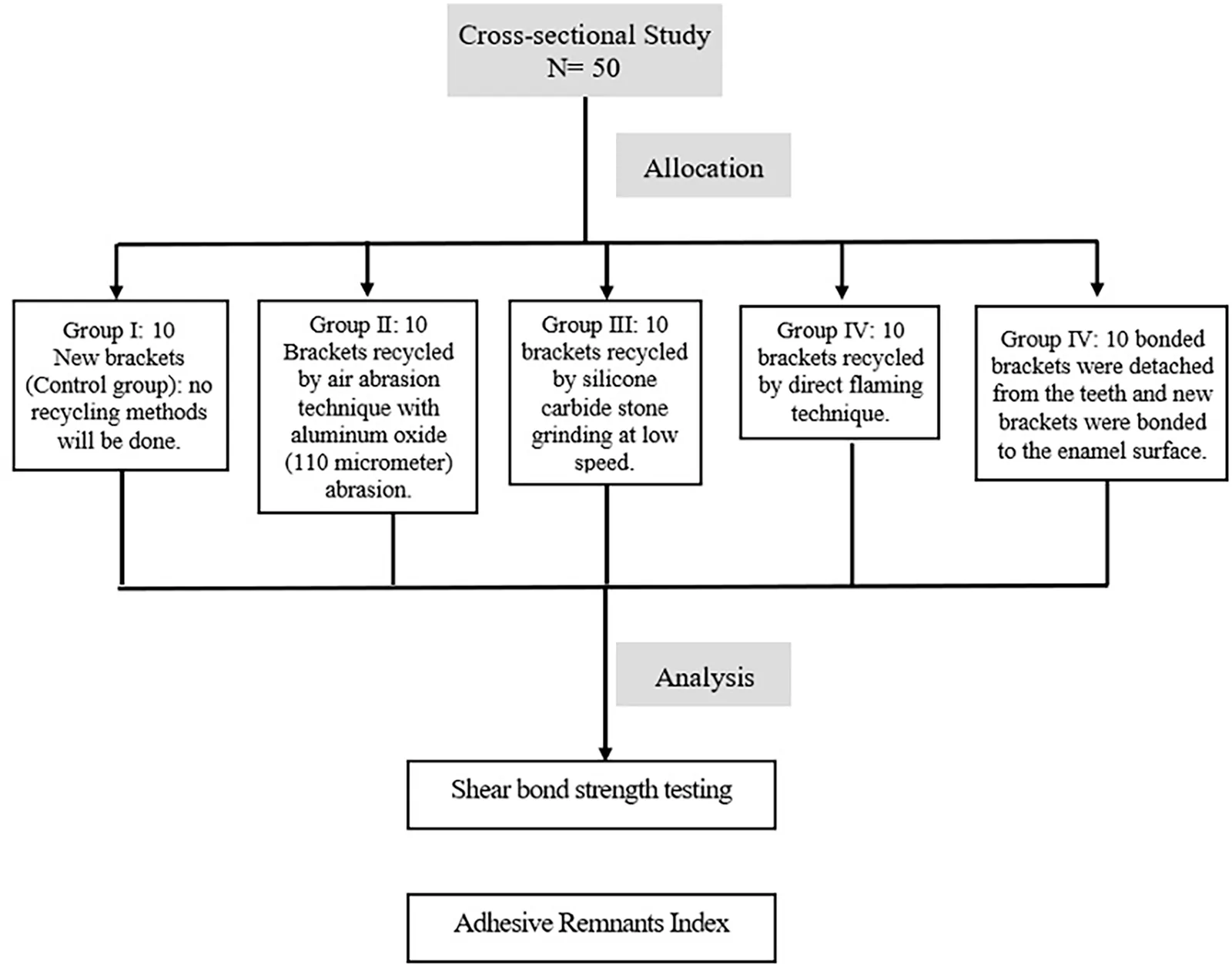
Showing the consort flow diagram.

Group I (control group) represents an initially bonded, brackets remained bonded to the enamel surface throughout storage and thermocycling and were not subjected to any debonding or rebonding cycle. In contrast, Groups II through V all underwent a complete debond–recycle/replace–rebond sequence before thermocycling and SBS testing (Table 1).

**Table 1:** Procedure table.

Step	Group I control	Group II air abrasion	Group III silicon carbide	Group IV direct flaming	Group V new brackets
1. Enamel preparation	Pumice, rinse	Pumice, rinse	Pumice, rinse	Pumice, rinse	Pumice, rinse
2. Initial bonding	Etch → primer → composite → LED cure	Etch → primer → composite → LED cure	Etch → primer → composite → LED cure	Etch → primer → composite → LED cure	Etch → primer → composite → LED cure
3. Storage	37 °C, 100% humidity, 24 h	37 °C, 100% humidity, 24 h	37 °C, 100% humidity, 24 h	37 °C, 100% humidity, 24 h	37 °C, 100% humidity, 24 h
4. Debonding	Not performed	Bracket removed	Bracket removed	Bracket removed	Bracket removed
5. Bracket treatment	–	Air abrasion (110 µm Al₂O₃, 80 psi, 25 s, 10 mm)	Silicon-carbide grinding (25,000 rpm, 25 s)	Direct flaming (1 cm, 5 s)	Bracket discarded; new bracket used
6. Rebonding	Not performed	Etch → primer → composite → LED cure	Etch → primer → composite → LED cure	Etch → primer → composite → LED cure	Etch → primer → composite → LED cure
7. Storage	–	37 °C, 100% humidity, 24 h	37 °C, 100% humidity, 24 h	37 °C, 100% humidity, 24 h	37 °C, 100% humidity, 24 h
8. Thermocycling	5–55 °C, 3,000 cycles	5–55 °C, 3,000 cycles	5–55 °C, 3,000 cycles	5–55 °C, 3,000 cycles	5–55 °C, 3,000 cycles
9. SBS testing	Universal testing machine, 1 mm/min	Universal testing machine, 1 mm/min	Universal testing machine, 1 mm/min	Universal testing machine, 1 mm/min	Universal testing machine, 1 mm/min

No recycling procedure was performed for the brackets group I (Control). The brackets remained bonded to the enamel and were tested without any reconditioning.

For brackets in Group II, the residual composite was removed using the intraoral sandblaster (Micro etcher II, Danville Materials, California, USA) with 110 µm aluminum oxide particles. The sandblasting was performed under a pressure of 80 psi for 25 s. The nozzle was positioned ten millimeters from the bracket base.

For specimens in Group III, residual composite was removed from the bracket base using a silicon carbide stone (Diacomp, 190 FG, Diaswiss, Nyon, Switzerland) mounted on a low-speed handpiece. The grinding was performed at a rotational speed of 25,000 rpm for approximately 25 s.

In Group IV, composite removal from the bracket base was performed using a direct flaming technique (butane micro-torch). A blow torch was used to apply medium heat at approximately 1 cm from the bracket base. Each bracket was exposed to the flame for 5 s, during which the residual adhesive was burned off. The surface of the bracket became glossy and exhibited a red-hot appearance, indicating sufficient thermal cleaning.

While in group V, the bonded brackets were detached from the teeth and new brackets were bonded to the enamel surface.

Standardized bonding was performed on all teeth using stainless-steel brackets (Roth 0.018″ slot; American Orthodontics, USA). The enamel surface was etched with 37% phosphoric acid for 30 s, rinsed and dried, then Ortho Solo™ primer (Ormco, USA) and Enlight™ light-cured composite (Ormco, USA) were applied. Brackets were positioned centrally on the buccal surface and light-cured with an LED unit (Woodpecker) at 1,200 mW/cm^2^ for 25 s. Specimens were stored in 100% humidity at 37 °C for 24 h prior to debonding.

Rebonding was performed using the same protocol as at the initial bonding (37% phosphoric-acid etch, Ortho Solo™ primer, Enlight™ composite, LED curing 25 s at 1,200 mW/cm^2^). After rebonding, all specimens were returned to 100% humidity at 37 °C for 24 h.

Thermocycling was then carried out between 5 °C and 55 °C for 3,000 cycles with a 30-s dwell time and 15-s transfer time to approximate intraoral temperature changes (Figure 2).

**Figure 2:**
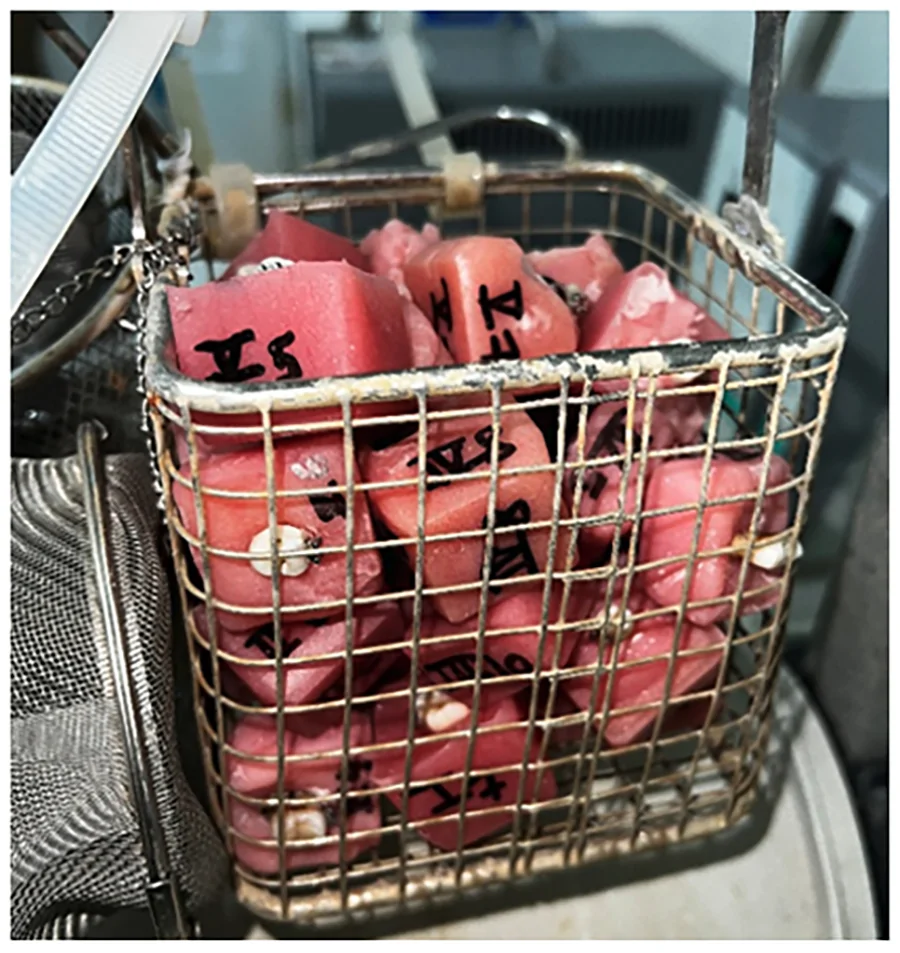
Acrylic-mounted premolar specimens labeled with waterproof marker and placed in a metal basket prior to thermocycling. Specimens were prepared for aging simulation using a thermocycler to replicate intraoral temperature variations between 5 °C and 55 °C.

 Shear bond strength testing was performed on a universal testing machine (YLE) using a chisel-shaped blade to apply a gingivo-occlusal load at the bracket–enamel interface. The crosshead speed was 1 mm/min until failure. The maximum force (*N*) was recorded and converted to MPa based on a bracket base area of 12.00 mm^2^ using the formula: SBS (MPa) = Load (*N*)/12.00 mm^2^ (Figure 3).

**Figure 3:**
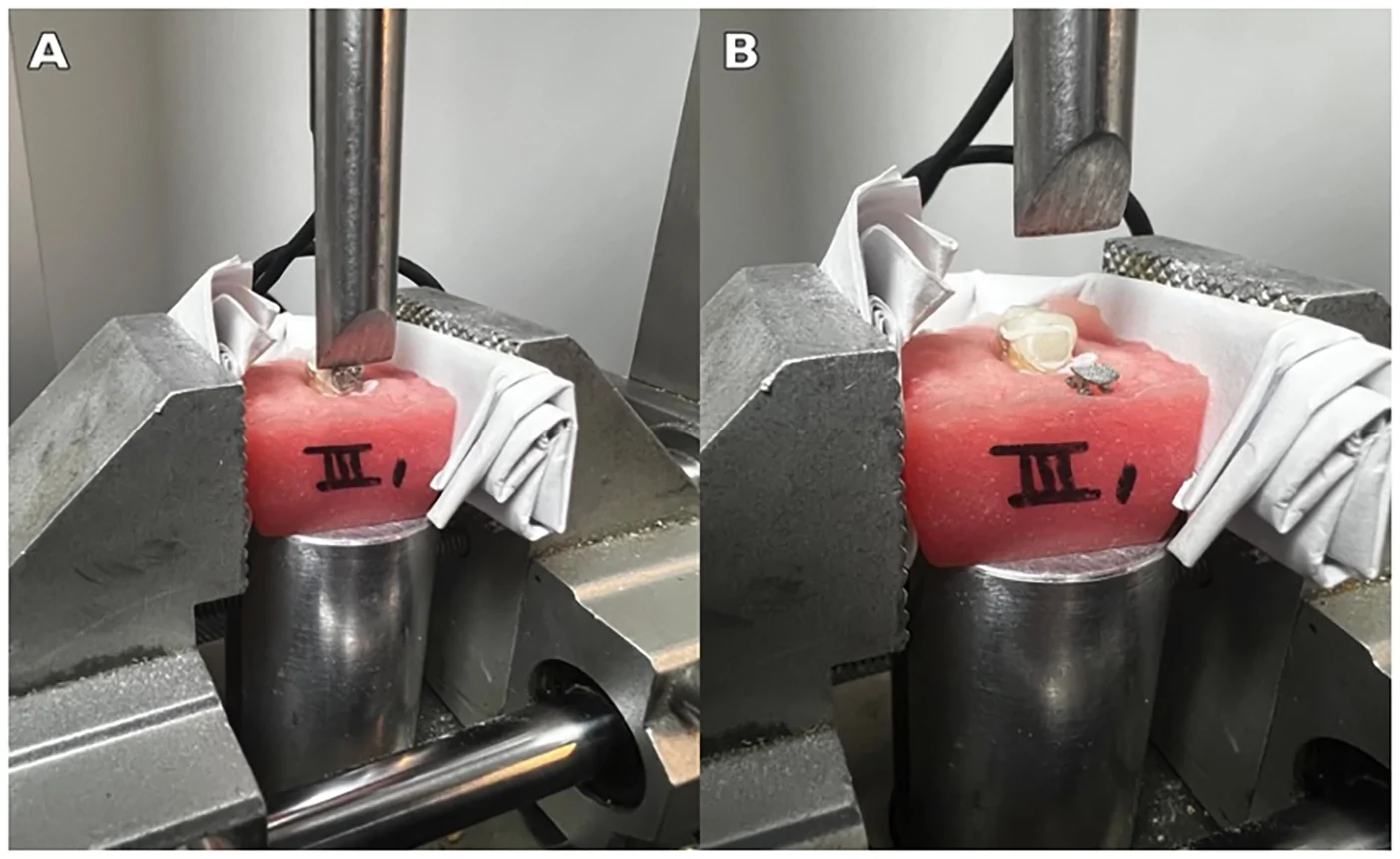
Shear bond strength (SBS) testing of a group III specimen. **(A)** The acrylic-mounted premolar is positioned in the universal testing machine with a chisel-edge blade aligned to apply gingivo-occlusal force at the bracket–tooth interface. **(B)** Post-debonding view showing the bracket and remaining adhesive dislodged from the enamel surface, confirming failure at the bonding interface.

Failure mode (Adhesive Remnant Index) was assessed under a stereomicroscope (×10). The ARI (0–3) was scored as follows: 0, no adhesive on enamel; 1, <50% coverage; 2, >50% coverage; 3, 100% coverage (Figure 4).

**Figure 4:**
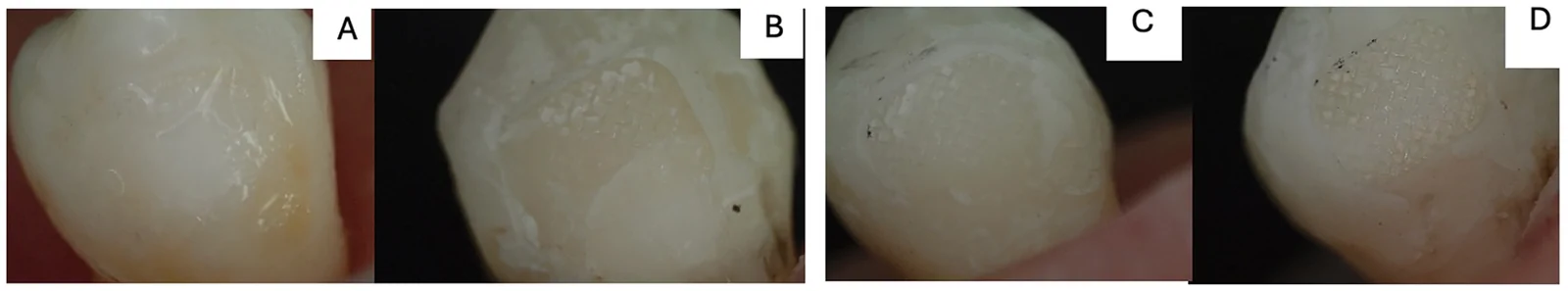
Examples of ARI scores. **(A)** score 0. **(B)** score 1. **(C)** score 2. **(D)** score 3.

To minimize measurement bias, teeth were randomly assigned to groups by a computer-generated list. SBS testing was performed by an operator blinded to group identity, and ARI scoring was carried out by a second examiner, also blinded. To assess intra-examiner reliability, ARI scoring was repeated two weeks later on ten randomly selected specimens.

Intra-examiner reliability for ARI scoring was assessed by repeating the evaluation on ten randomly selected specimens two weeks after the initial assessment. Agreement was quantified using weighted kappa (*κ*w), with quadratic weighting applied to account for the ordinal nature of the scale.

Statistical analysis was conducted with non-parametric tests. Continuous variables (SBS) were summarized as mean ± SD. Group comparisons were performed using the Kruskal–Wallis test (*α* = 0.05) with Mann–Whitney *post-hoc* tests for pairwise analysis. ARI distributions were compared descriptively across groups.

### Statistical analysis

All statistical analyses were performed using IBM SPSS version 28. Descriptive statistics were used to summarize the continuous variables using means and standard deviations. A nonparametric Kruskal–Wallis test was used to assess differences in SBS distributions across the five groups. Pairwise comparisons were conducted using the Mann–Whitney test. All statistical tests were two-sided, and a *p*-value <0.05 was considered statistically significant.

## Results

This study was conducted on 50 extracted human premolars, which were randomly assigned to 5 groups of ten teeth each, according to the recycling techniques used. In the control group (Group 1), the bonded brackets remained attached until shear testing. In groups 2, 3, and 4, the recycling techniques used were the air abrasion technique with aluminum oxide (110 micrometer) abrasion, silicon carbide stone grinding at low speed, and the direct flaming technique, respectively. In group 5, the bonded brackets were detached from the teeth, and new brackets were bonded to the enamel surface.

### Descriptive statistics

The Kruskal–Wallis test revealed a statistically significant difference in SBS distributions across groups (H(4) = 29.669, *p* < 0.001, *η*^2^ = 0.570). The mean values ranged between 3.27 ± 1.55 and 6.44 ± 3.41.

Table 2 presents the results of all 10 pairwise Mann–Whitney U comparisons with Holm-adjusted *p*-values and effect sizes. Six comparisons remained statistically significant after correction. The control group differed significantly from the air abrasion group (Holm-adjusted *p* = 0.001, *r* = 0.727) and from the new brackets group (Holm-adjusted *p* = 0.006, *r* = 0.625). The air abrasion group differed significantly from both the silicon-carbide grinding group (Holm-adjusted *p* = 0.0003, *r* = 0.820) and the direct flaming group (Holm-adjusted *p* = 0.001, *r* = 0.744). The new brackets group likewise differed significantly from the silicon-carbide grinding group (Holm-adjusted *p* = 0.0005, *r* = 0.786) and the direct flaming group (Holm-adjusted *p* = 0.002, *r* = 0.693). No significant difference was found between the air abrasion group and the new brackets group (Holm-adjusted *p* = 0.545), confirming comparable SBS between these two groups. The control group did not differ significantly from either the silicon-carbide grinding group (Holm-adjusted *p* = 0.017) or the direct flaming group (Holm-adjusted *p* = 0.063) after correction, and no significant difference was observed between the grinding and flaming groups (Holm-adjusted *p* = 0.970) (Figure 5).

**Table 2:** Descriptive statistics of shear bond strength in different recycling techniques groups.

Group	*n*	Mean ± SD (MPa)	Median (MPa)	IQR (MPa)	Min	Max	*P* value
I — Control	10	4.30 ± 0.43	4.38	3.95–4.56	3.75	5.08	< 0.001
II — Air abrasion	10	6.22 ± 1.30	6.17	5.04–7.14	4.41	8.25	
III — Silicon carbide	10	3.41 ± 0.86	3.42	2.72–4.21	2.00	4.50	
IV — Direct flaming	10	3.27 ± 1.55	3.29	3.04–4.04	0.25	5.50	
V — New brackets	10	6.44 ± 3.41	5.21	5.00–6.33	4.00	15.75	

Descriptive statistics were used to calculate means and standard deviations.

*p*-values are Holm-adjusted for 10 pairwise comparisons.

*r* *=* rank-biserial correlation coefficient.

**Figure 5:**
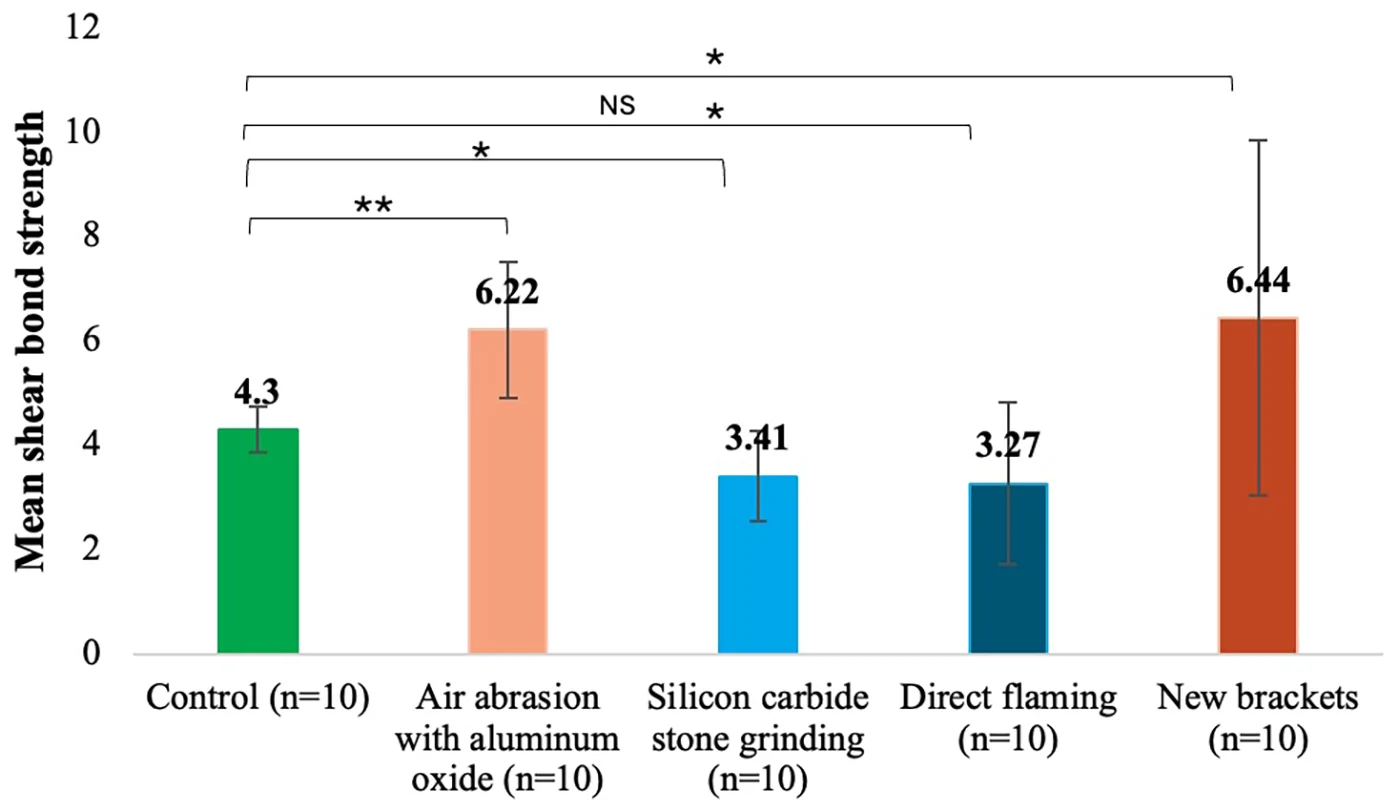
Means shear bond strength among groups.

Table 3 presents the results of all 10 pairwise Mann–Whitney U comparisons with Holm correction applied across the full family of tests. Six comparisons remained statistically significant after correction, all demonstrating large effect sizes (*r* = 0.625–0.820). Both the air abrasion group and the new brackets group produced significantly higher SBS than the control group (*p* = 0.001, *r* = 0.727; and *p* = 0.006, *r* = 0.625, respectively). The air abrasion group also differed significantly from both the silicon-carbide grinding group (*p* = 0.0003, *r* = 0.820) and the direct flaming group (*p* = 0.001, *r* = 0.744), as did the new brackets group compared to both (*p* = 0.0005, *r* = 0.786; and *p* = 0.002, *r* = 0.693, respectively).

**Table 3:** Pairwise comparison.

Comparison	*U*	*Z*	*p*	Holms	*r*
Air abrasion vs. silicon carbide	98.5	3.666	0.0003	0.005	0.820*
New brackets vs. silicon carbide	3.5	−3.515	0.0005	0.006	0.786*
Air abrasion vs. direct flaming	94.0	3.326	0.0010	0.006	0.744*
Control vs. air abrasion	7.0	−3.250	0.0013	0.007	0.727*
New brackets vs. direct flaming	9.0	−3.099	0.0022	0.008	0.693*
Control vs. new brackets	13.0	−2.797	0.0057	0.010	0.625*
Control vs. silicon carbide	82.0	2.419	0.017	0.013	0.541
Control vs. direct flaming	75.0	1.890	0.063	0.017	0.423
Air abrasion vs. new brackets	58.5	0.643	0.545	0.025	0.144
Silicon carbide vs. direct flaming	49.0	−0.076	0.970	0.050	0.017

*Statistically significant.

Four comparisons were not significant after Holm correction. The control group did not differ significantly from either the silicon-carbide grinding group (*p* = 0.017) or the direct flaming group (*p* = 0.063) once the correction was applied. No significant difference was found between the air abrasion group and the new brackets group (*p* = 0.545, *r* = 0.144), confirming statistically comparable bond strength between these two groups. Similarly, silicon-carbide grinding and direct flaming did not differ from each other (*p* = 0.970, *r* = 0.017), indicating that these two recycling methods produced equivalent and comparably reduced bond strengths.

Taken together, the pairwise results identify a clear two-tier pattern; the air abrasion and new brackets groups formed a high-SBS cluster, significantly superior to the low-SBS cluster of silicon-carbide grinding and direct flaming, while the control group occupied an intermediate position that was not significantly distinguishable from either cluster after correction.

Notably, the SBS values for the silicon-carbide grinding group were based on the manufacturer bracket-base area of 12.00 mm^2^, which may not accurately reflect the effective bonding area after mechanical grinding. If grinding reduced the functional base area, the true SBS in this group may be lower than reported; if surface irregularities increased the contact perimeter, it may be slightly higher. This uncertainty could vary the absolute MPa values for this group.

In Table 4, presents the complete ARI score distribution across all groups. In the recycled groups, the majority of specimens recorded an ARI score of 3: 7 of 10 in the air abrasion group, 10 of 10 in the silicon-carbide grinding group, and 8 of 10 in the direct flaming group, indicating that virtually all adhesive remained on the enamel surface after debonding. The control group showed a more varied distribution, with 1,2,3 and 4 specimens recording ARI scores of 0, 1, 2, and 3, respectively. The new brackets group similarly showed mixed scores, with 0,3,3 and 4 specimens at ARI scores of 0, 1, 2, and 3 (Figure 6).

**Table 4:** Frequencies of different ARI values in different recycling techniques groups.

ARI SCORES	ARI 0	ARI 1	ARI 2	ARI 3
Groups				
1	1	2	3	4
2	0	1	2	7
3	0	0	0	10
4	0	0	2	8
5	0	3	3	4

**Figure 6:**
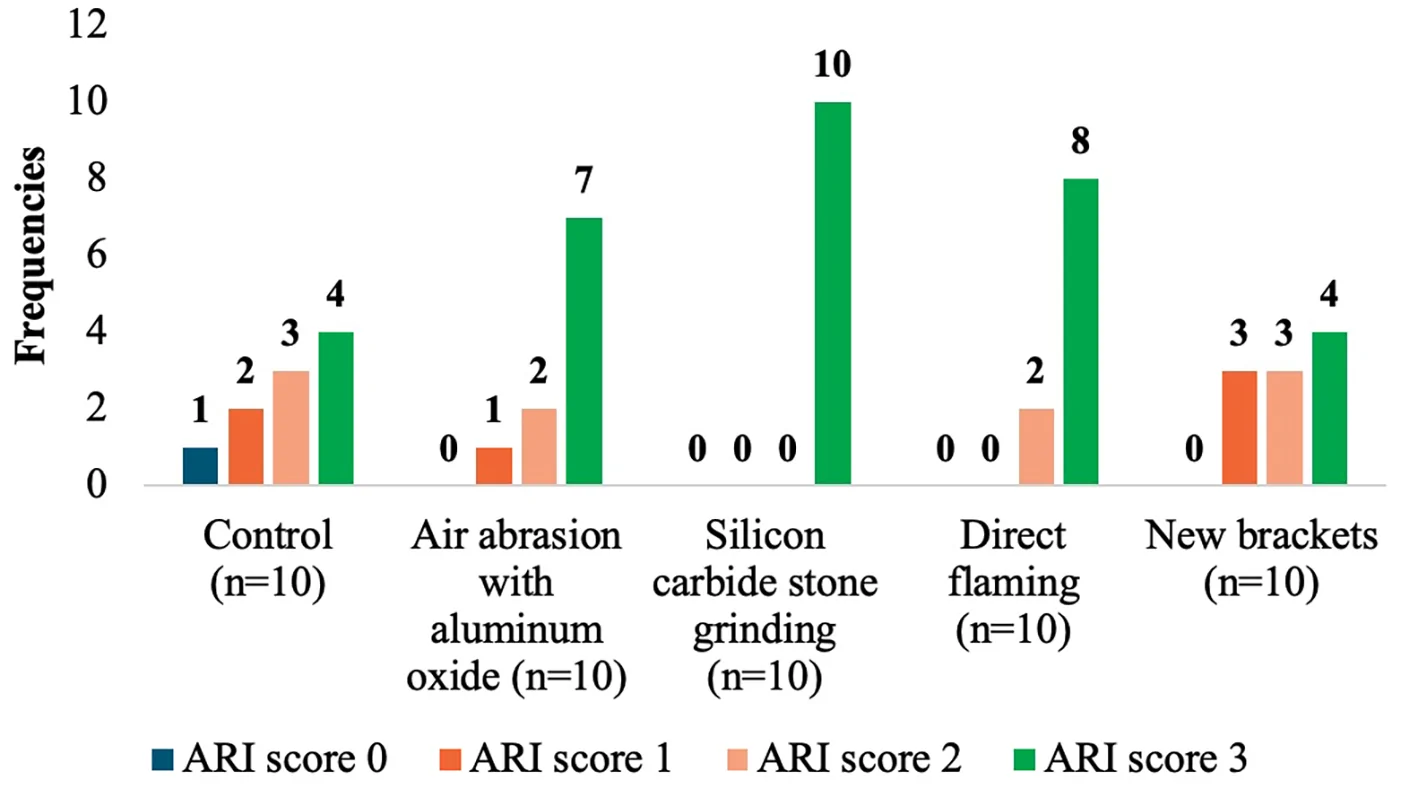
Frequencies of different ARI values in different recycling techniques groups.

Intra-examiner reliability for ARI scoring was substantial (weighted *κ*w = 0.76; 95% CI: 0.39–1.00), with 7 of 10 re-scored specimens showing exact agreement and the remaining 3 differing by only one category.

## Discussion

Among the pragmatic aspects of fixed orthodontics, a consistent connection between the bracket base and the enamel is the key to treatment efficiency and cost-effectiveness. Debonded bracket recycling can potentially decrease clinical waste and cost; however, it must be ensured that the reconditioning process maintains bond performance without compromising enamel integrity upon removal. Air abrasion with aluminum oxide is frequently recommended in the literature because it is expected to increase base roughness and micromechanical retention, whereas stone grinding has been reported to risk mesh damage and direct flaming to cause thermal alteration of the base, both of which may diminish retention (10, 11).

In the current experiment, stainless-steel brackets were reconditioned using three chairside strategies of airborne-particle abrasion using 110 µm Al 2 O 3 at 80 psi and 25 s; silicon-carbide stone grinding (Diacomp 190 FG, Diaswiss) at 25,000 rpm and 25 s; and direct flaming at 1 cm and 5 s; and compared with previously bonded non-recycled brackets and control. Standard bonding was done (37% phosphoric-acid etch, Ortho Solo TM primer, Enlight TM composite, LED-curing, 25 s, 1,200 mW/cm 2), and all were thermocycled (5 55 C, 3,000 cycles) before testing, in line with contemporary *in-vitro* protocols (8, 9, 12).

Our findings showed that air abrasion produced SBS values comparable to new brackets, whereas grinding and flaming yielded significantly lower values and did not differ from each other. A plausible mechanical explanation is that airborne-particle abrasion removes residual resin and may re-texture the bracket base, thereby improving aperture accessibility and surface area available for resin penetration and micromechanical interlocking at rebond (8, 10). By contrast, rotary grinding could potentially plane mesh peaks and reduce undercuts, limiting resin-tag formation, while direct flaming may burn off adhesive but could simultaneously induce oxidation or surface-energy changes that weaken the resin–metal interface (5, 12). Because no surface characterization (e.g., SEM or profilometry) was performed in the present study, these mechanistic explanations remain hypothetical and require confirmation by future investigations incorporating direct surface analysis.

The bracket-base area was fixed at 12.00 mm^2^ for all groups based on the manufacturer's specification (American Orthodontics, Sheboygan, WI, USA) and in accordance with the approach used in the majority of comparable *in-vitro* studies. Although recycling procedures may theoretically alter bracket-base morphology and effective bonding area, no direct measurement of individual bracket-base areas was performed in this study. The use of a standardized area across all groups facilitates direct inter-group comparison and consistency with published literature, while recognizing that this represents a potential source of systematic error in the grinding group specifically.

Since the SBS values for the grinding group were calculated using the manufacturer-stated bracket-base area of 12.00 mm^2^ applied uniformly across all groups. Silicon-carbide grinding may alter the effective bonding area of the bracket base, meaning the reported MPa values for this group carry an inherent measurement uncertainty. While this does not affect the rank-based statistical comparisons, it should be taken into account when comparing the absolute SBS values of the grinding group with those of the other groups or with values reported in the literature.

Failure-mode analysis provides additional context. The predominance of ARI score 3 in the recycled groups indicates that failure occurred predominantly at or near the bracket–adhesive interface, leaving a substantial amount of adhesive on the enamel surface after debonding. This pattern may be considered clinically preferable from an enamel-risk perspective, as adhesive coverage could reduce the likelihood of direct enamel damage during debonding; however, it is important to note that enamel integrity was not directly assessed in the present study, and ARI scoring serves only as an indirect indicator of the failure location. Any remaining adhesive should be removed carefully using fine tungsten-carbide burs under water cooling and light pressure (13). In contrast, the control and new-bracket groups showed more mixed ARI distributions, reflecting greater variability in failure location when the bracket base is untreated or pristine.

These results align closely with earlier work. Salama et al. found that sandblasted rebonded brackets exhibited higher SBS values and a tendency toward higher ARI scores, suggesting that abrasive reconditioning may restore or improve bond performance; whether this translates into enamel protection was not directly verified in that study either (7). The similarity between our air-abrasion and new-bracket groups likely reflects protocol variables, including adhesive system, bracket design, and particle size (50 µm versus 110 µm), rather than a categorical superiority of recycled brackets.

Tavares et al. (2006) found that control, air-abraded recycled, and new brackets were all similar. However, their control was lower because they used different methods, such as no thermocycling, chemically cured resin, and a slower crosshead speed (8).

Halwai et al. (2012) likewise found that sandblasting was better than flaming and bur grinding (5); Ismail et al. (2013) confirmed sandblasting as the most effective office reconditioning across base designs (1); and Zafarmand et al., showed that 90-µm Al_2_O_3_ sandblasting can achieve SBS comparable to initially bonded brackets (13). Venugopal et al, demonstrated sandblasting is better than flaming with no significant difference from new brackets (9). Kachoei et al, indicated that repeated Al_2_O_3_ sandblasting maintains favorable SBS and performs at least comparably to CO_2_ laser across re-bond cycles (8).

Johns et al. (2020) reported air abrasion to be better than flaming with a crosshead speed of 1 mm/min, which agrees with our ranking (14). Pereira et al. (2022) demonstrated that sandblasting was substantially greater as compared to superficial or thorough greenstone grinding, and as such, aggressive grinding can flatten the mesh and decrease retention (15).

From a clinical standpoint, these results indicate that airborne-particle abrasion, applied with standardized parameters, represents the most effective chairside method for reconditioning debonded metal brackets, yielding SBS values comparable to new brackets and a failure pattern characterized predominantly by adhesive retention on enamel (ARI score 3). This failure distribution may indicate a lower risk of direct enamel damage at debonding, though this inference is indirect and could not be confirmed without dedicated enamel surface assessment. Stone grinding and direct flaming appear less suitable as stand-alone recycling methods; if minimal grinding is required to eliminate adhesive nodules, it should be followed by a brief air-abrasion pass to restore surface texture.

This study has typical limits of *in-vitro* research: the absence of saliva, pH fluctuations, cyclic mastication, and long-term intraoral aging; the use of a single bracket brand and adhesive system; one recycling cycle; and no SEM/profilometry to visualize base alteration. Nevertheless, the inclusion of a standardized bonding protocol, thermocycling, a previously bonded non-recycled control, and a new-bracket comparator strengthens interpretation and comparability with existing literature.

Moreover, the use of the manufacturer-stated bracket-base area (12.00 mm^2^) as a fixed denominator for SBS conversion across all groups represents a particular limitation for the silicon-carbide grinding group. Mechanical grinding may alter bracket-base morphology and reduce or redistribute the effective bonding area, introducing a degree of systematic measurement error into the absolute MPa values reported for this group. As a result, the SBS values for the grinding group should be interpreted with caution, and direct comparison of absolute values with other groups or with literature figures using individually measured base areas may not be fully equivalent. Future studies should consider measuring bracket-base area individually following recycling, particularly for mechanically altered surfaces, to allow more accurate SBS conversion.

## Conclusion

Within the constraints of this *in-vitro* study, the bracket recycling technique significantly influenced shear bond strength. Airborne-particle abrasion with aluminum oxide produced bond strengths comparable to those of new brackets, while silicon-carbide stone grinding and direct flaming resulted in significantly lower values.

Air abrasion was the most consistent reconditioning technique, matching the performance of unused brackets and yielding a predominantly adhesive-retentive failure pattern (ARI score 3), which may indicate a lower risk of enamel damage at debonding. However, since enamel integrity was not directly evaluated in this study, claims regarding enamel protection cannot be established from these data alone. Grinding and flaming did not perform adequately as stand-alone recycling methods and should be used with caution. Future studies incorporating direct surface characterization (SEM) and enamel assessment would strengthen the mechanistic and clinical inferences drawn here.

## Data Availability

The datasets presented in this study can be found in online repositories. The names of the repository/repositories and accession number(s) can be found in the article/Supplementary Material.
